# Metabolomics in Psychiatric Disorders: What We Learn from Animal Models

**DOI:** 10.3390/metabo10020072

**Published:** 2020-02-17

**Authors:** Elke Humer, Thomas Probst, Christoph Pieh

**Affiliations:** Department for Psychotherapy and Biopsychosocial Health, Danube University Krems, 3500 Krems, Austria; thomas.probst@donau-uni.ac.at (T.P.); christoph.pieh@donau-uni.ac.at (C.P.)

**Keywords:** animal models, biomarkers, metabolomics, psychiatric disorders

## Abstract

Biomarkers are a recent research target within biological factors of psychiatric disorders. There is growing evidence for deriving biomarkers within psychiatric disorders in serum or urine samples in humans, however, few studies have investigated this differentiation in brain or cerebral fluid samples in psychiatric disorders. As brain samples from humans are only available at autopsy, animal models are commonly applied to determine the pathogenesis of psychiatric diseases and to test treatment strategies. The aim of this review is to summarize studies on biomarkers in animal models for psychiatric disorders. For depression, anxiety and addiction disorders studies, biomarkers in animal brains are available. Furthermore, several studies have investigated psychiatric medication, e.g., antipsychotics, antidepressants, or mood stabilizers, in animals. The most notable changes in biomarkers in depressed animal models were related to the glutamate-γ-aminobutyric acid-glutamine-cycle. In anxiety models, alterations in amino acid and energy metabolism (i.e., mitochondrial regulation) were observed. Addicted animals showed several biomarkers according to the induced drugs. In summary, animal models provide some direct insights into the cellular metabolites that are produced during psychiatric processes. In addition, the influence on biomarkers due to short- or long-term medication is a noticeable finding. Further studies should combine representative animal models and human studies on cerebral fluid to improve insight into mental disorders and advance the development of novel treatment strategies.

## 1. Introduction

Psychiatric disorders are a major burden for public health worldwide, affecting patients, society, and nations as a whole [1]. There is a high need for the development of biomarkers that could help improve diagnosis, treatment monitoring and prediction of treatment response to improve patient outcomes [2]. However, research in this area has still not lived up to its potential. One main reason behind this is that psychiatric illnesses are thus far classified using diagnostic concepts which could be subjective [3]. Therefore, the incorporation of biomarkers into current clinical methods might help to generate a classification system for mental health disorders that can be linked to the underlying dysfunctional pathways, as proposed by the National Institute of Mental Health Research Domain Criteria (RDoC) project [4]. Together with an improved understanding of the pathways underpinning mental disorders, the discovery of novel biomarkers might facilitate more targeted treatments [3].

In general, biomarkers are endogenous compounds that are found to be changed responding to a tested stimulus. As such, biomarkers are characteristics that are objectively measured and evaluated as indicators of normal biological factors, pathogenic processes, or pharmacological responses to therapeutic interventions [5]. At best, a biomarker should reflect the underlying disease characteristics, be available within a short time frame, and at an affordable cost, and provide measurements with high accuracy and reproducibility [3]. Considering these requirements, the traditional biomarkers available in psychiatry, including those from brain imaging analyses, psychiatric tests, and electrophysiological responses, typically show several limitations. Therefore, there is a need to discover novel biomarkers that provide information about the underlying disease, while being economically affordable.

Recent advances in analytical technology enable bioinformatics studies on genes, transcripts, proteins, and metabolites, generically known as “omics technologies” [6,7]. The metabolome represents the “ome” which is inevitably closest to the phenotype [8]. As metabolites are the end products and by-products of complicated biosynthetic and catabolism pathways, metabolomics, is a powerful technique to investigate the phenotypic changes caused by exogenous stimuli more predictively than other omics approaches [6,7,8]. In this regard, the study of metabolites in a large-scale manner is nowadays considered one of the most informative representations of biological functions, considering that these molecules actually carry out or respond to most processes of the body [2,3]. Thus, metabolite profiling seems promising to reveal early biochemical changes in disease and hence provides an opportunity to develop predictive biomarkers that can trigger earlier interventions [9]. Recent applications of metabolomics cover widespread areas, including disease diagnosis and screening, therapeutics toxicity and efficacy assessment, drug discovery and development, patient stratification and monitoring of patient response to treatment [2,10].

In recent years, molecular signatures for psychiatric disorders in humans were detected in the circulating blood. However, as psychiatric disorders appear to be generated in the brain, analysis of brain tissues might offer the best insight into the underlying pathophysiological mechanisms [3]. Unfortunately, obtaining such samples from living humans is challenging due to the high invasiveness and expense of lumbar puncture and brain biopsy [11]. Therefore, human brain tissues are typically only available at postmortem [3]. Thus, animal studies might offer a valuable option to gain a deeper insight into the mechanisms underlying psychiatric disorders. Combining the analysis of brain tissues with blood or urine samples also helps to identify whether the effects of diseases can be seen throughout the whole body. This is in agreement with the whole-body concept of psychiatry, suggesting that the brain is integrated into virtually all physiological functions of the body, which is assumed to be reflected in the composition of blood proteins and other bioactive molecules [3,12]. Thus, animal models might offer ways to gain direct insights into the mechanisms underlying disorders as well as drugs used in their treatment. For further application in human medicine, however, the use of blood or urine are more amenable to clinical studies since they can be taken from living patients in a minimally invasive way during progressive stages of the disease or following treatment [3].

Metabolomic signatures have already been reported for several diseases including major depressive disorder, bipolar disorder, schizophrenia, and drug addiction [2,13]. The following sections provide examples of how the use of advanced metabolomic platforms in combination with animal models permits a global and integrated analysis of biochemical abnormalities and metabolic changes occurring in psychiatric disorders as well as their treatment.

The aim of this review is to provide an overview of the application of metabolomics using animal models in studies in psychiatry. It comprises examples of metabolomics research on animal models of psychiatric diseases, highlights the potential of these models for the study of psychiatric diseases, but also the limitations, which should be kept in mind when using animal models.

## 2. Examples of Using Metabolomics in Animal Models for the Study of the Pathogenesis of Psychiatric Diseases

A literature search was conducted using the public search generators Web of Science, ScienceDirect, and Scopus. Research articles in scientific journals on experiments using animal models of psychiatric disorders published in English were primarily considered. The search was conducted on 13 December 2019, with no limitations on the publication date. The terms used in the literature search were “metabolomics” AND “psychiatric disorder” OR “mental disorder” OR “depression” OR “bipolar disorder” OR “anxiety” OR “schizophrenia” OR “addiction” OR “addictive disorder”. Studies using animal models to unravel the pathophysiology of mental disorders or their treatment were selected. A summary of relevant studies based on animal models to unravel the pathophysiology of mental disorders including pathways involved in mental disorders, as well as possible biomarkers, in a variety of psychiatric disorders are represented in the following sections.

### 2.1. Metabolomics in Depression Models

Major Depressive Disorder (MDD) is a prevalent psychiatric disorder, affecting 4.4% of the world’s population [14,15]. This serious mental illness is associated with a varied prognosis, chronic course, and duration of illness with reduced quality of life [16]. There are no validated biomarkers for MDD, and the current diagnostic categories are mainly based on self-reports, measurement-based scales, with a lack of understanding of the molecular blood testing compared to other diseases [17]. The current practice to explore demographic features, illness characteristics, psychosocial functioning, and social factors has proven to be of limited utility due to the knowledge gap regarding cellular and molecular pathophysiology [18]. Therefore, the exploration of the underlying biological factors that drive MDD has been considered to be more applicable as biomarkers for guiding personalized medicine, as they are objective and can be measured externally [19].

As summarized in Table 1, animal metabolomic studies were mainly applied to brain-derived material of rodents. Animal models of depression typically rely on chronic stress paradigms, where normal rodents are subjected to a series of physical stressors, such as restraint, footshock or cold temperature [20]. These models were employed based on the observation that stress and emotional losses are potent risk factors of depression [21]. A key issue in studying the mechanisms of mental disorders is the selection of appropriate sampling material. On the basis of rat models of depression, MDD seems to be especially associated with metabolic dysfunction in the hippocampus and prefrontal cortex [15]. More specifically, animal depression studies revealed that amino acid (AA) metabolism is disturbed in various brain regions, but also in the plasma of depressed individuals. Especially the glutamate-γ-aminobutyric acid (GABA)-glutamine cycle, which has been implicated to be related to the pathogenesis of MDD, due to its key role in maintaining the supply of neurotransmitters [22]. Overall, the glutamatergic system plays an important role in the pathophysiology of mood disorders, such as depression, where glutamate disturbances in the brain have been linked to the neuroplasticity hypothesis of MDD [23]. In this regard, the downregulation of glutamine in the brain, which represents the most abundant free AA in mammals, was a common observation in all investigated rodent models of depression (Table 1).

Next to changes related to the glutamate-GABA-glutamine-cycle, MDD-related alterations included AA metabolites involved in the synthesis of monoamine and AA neurotransmitters (tryptophan, tyrosine, glycine, aspartate), proteinogenic branched-chain AA (leucine, isoleucine), oxaloacetate-derived AA (threonine, methionine), which are mainly involved in the biosynthesis of proteins and energy production via gluconeogenesis. Additionally, energy metabolism dysfunction has been related to the pathogenesis of multiple psychiatric disorders, such as MDD. Indeed, as summarized in Table 1 several energy metabolism-related metabolites (palmitic acid, stearic acid, pyruvic acid) were altered in animal models of depression [27]. In addition, changes in catecholaminergic pathways in the prefrontal cortex have been observed to be responsible for discriminating metabolic profiles of depressed compared to healthy mice [1].

Although rodent models are most commonly used in the study of human illnesses, higher primates like macaques seem to be especially appropriate as an animal model for psychiatric disorders. Cynomolgus macaques represent some of the phylogenetically closest relatives of humans. Thus, they have been increasingly used in the evaluation of new drugs, including antidepressants [28]. Compared to traditional rodent models, the naturally occurring depressed cynomolgus monkeys are screened from a more natural environment. Therefore, these models are expected to closely resemble the etiological development of MDD [15]. In a recent study, the metabolomic profile of cerebrospinal fluid in a naturally occurring depressive model in macaques was compared to healthy controls [15]. Depressed macaques showed perturbations of fatty acid biosynthesis and AA metabolism in the cerebrospinal fluid. Furthermore, eight short-chain fatty acids (SCFA) and AA were also altered in the serum. Thus, perturbations in SCFA and AA in the central, as well as peripheral system, seem to be involved in the pathogenesis of depression. Studies conducted on human cerebral fluid or plasma samples, also observed pronounced differences in AA concentrations between healthy and depressed individuals [22,29]. Moreover, alterations of five metabolites involved in the ABC transport system were observed in depressed monkeys compared to their healthy counterparts. As ABC transporters represent an integral part of the blood-brain barrier, disturbances in this system might reduce the ability to selectively restrict the passage of substances from the blood to the brain [30], which has been linked to the emergence of neuropsychiatric diseases, such as depression, schizophrenia, and epilepsy [31,32].

In recent years, increasing evidence pinpoints to the central role of the gut microbiota in the pathogenesis of depression through the microbiota-gut-brain axis. In this regard, Deng et al. [29] also observed alterations in microbial metabolites (i.e., methanamine, benzeneacetic acid) in a depression model of macaques, indicating a contribution of the intestinal microbiome in MDD. Additionally, rodent models were used to improve the understanding of the microbiota-gut-brain axis. Yu et al. [33], for instance, combined fecal microbiological analysis with metabolomic analysis of the feces of depressed rats compared to healthy ones. Changes in fecal AA, fatty acid, bile acids, hypoxanthine, and stercobilins went along with perturbations in the gut microbiome, suggesting that MDD disturbs the gut microbiota as well as metabolic homeostasis.

### 2.2. Metabolomics in Anxiety Models

Anxiety disorders range among the most prevalent psychiatric disorders [34]. Studies in rodents were carried out to unravel the affected pathways for anxiety disorders and to identify biomarkers (Table 2). To investigate the metabolomic underpinnings of trait anxiety, mouse models with different anxiety-related behavior were generated by selective inbreeding based on the response to a weak emotional stressor such as exposure to an open arm of the elevated plus-maze [35,36], a model considered to be a robust system for the study of genetically inherited anxiety traits [37]. These studies point to the role of oxidative stress, alterations in AA and energy metabolism (i.e., mitochondrial regulation) and neurotransmission [35,36].

Recently, metabolomics approaches were also conducted on fearful dogs (Table 2). Compared to classical rodent models, dogs share comparable lifestyle and environmental factors like humans and are even genetically and physiologically closer to humans [38]. The study revealed changes in glutamine metabolism (i.e., higher plasma glutamine and γ-glutamyl-glutamine levels in fearful vs. non-fearful dogs). The essential AA glutamine serves not only as a precursor for the neurotransmitter glutamate, the most abundant and primary excitatory neurotransmitter in the central nervous system, but also for the neurotransmitter GABA [39], for antioxidant glutathione [40], protein, nucleotide and nucleic acid synthesis [41], as a substrate for gluconeogenesis [41] and ammonia genesis [42]. Glutamine actions in the brain are mostly mediated by the degradation of glutamine to glutamate as a part of the glutamate-glutamine cycle [39]. Glutamate represents the primary and most abundant excitatory neurotransmitter in the central nervous system. Thus, sufficient function of the glutamate-glutamine cycle is of utmost importance for appropriate glutaminergic neurotransmission [43]. As glutamate plays an important role in fear conditioning [44,45], dysfunction of this cycle has been suggested to be involved in different forms of anxieties, thus playing a central role in a wide spectrum of neuropsychiatric disorders, such as schizophrenia, depression and post-traumatic stress disorder [38]. Indeed, as summarized in Table 1, Table 2, Table 3 and Table 4, disturbances in glutamate concentration is a common finding in animal studies of different psychiatric disorders.

### 2.3. Metabolomics in Models of Schizophrenia

Schizophrenia is a severe chronic psychiatric disorder, characterized by hallucinations, delusions, social withdrawal and cognitive deficits, which affect approximately 1% of the population worldwide [46,47]. The study of the underlying pathophysiology of schizophrenia is complicated by its complex presentation and symptom heterogeneity [13]. Additionally, varying treatment options impede metabolomic studies. To avoid these confounders, ideally, drug-naive individuals should be examined. However, studies on postmortem brain tissue in humans are typically conducted on individuals who have been treated with varying lifetime antipsychotic medication doses [48,49]. Animal models used in schizophrenia research are widely based on rodents treated with N-methyl-D-aspartate (NMDA) receptor antagonists [50], which has been shown to cause positive as well as negative symptoms of schizophrenia [51]. Studies in rodent models of schizophrenia (Table 3) revealed that not only neurotransmitters but also metabolites involved in energy metabolism were altered by schizophrenia [52]. More specifically, a strongly interconnected pathway of downregulated glutamate synthesis and disturbed TCA cycle was identified, revealing biochemical connections among the proposed theories of disturbances on the glutamate neurotransmitter system and energy metabolism in schizophrenia [53,54]. As described above, the glutamate system is known to be involved in a wide spectrum of cerebral functions. Excitation by glutamate can cause pathological symptoms, such as mania or panic [43]. Additionally in humans, hyperactivity of the glutamate system has been associated with schizophrenia [55]. Next to changes in pathways involved in neurotransmission, alterations in arginine and proline metabolism, as well as disruptions in a series of purine reactions, were suggested to contribute to the compromised prefrontal cortex dysfunction and cognitive deficits, which are characteristics of schizophrenia [56].

### 2.4. Metabolomics in Addictive Disorders

Substance use disorders are highly prevalent, disabling and associated with various physical health problems and psychiatric disorders, such as MDD, BD, phobias and personality disorders [57,58]. Metabolomic approaches in addictive disorders are aimed at improving the understandings of addictive mechanisms, estimating potential biomarkers to confirm drug abuse and to identify therapeutic targets [2].

In contrast to most animal models in psychiatry research, which have been criticized for their limited translational value to the clinical situation, animal addiction models are considered to closely resemble the molecular and neurobiological changes that occur in drug-addicted humans [59]. Several studies have been conducted in animal models of drug addiction (Table 4), with the main focus on the involvement of brain function in addictive behaviour. These studies were mainly conducted on samples derived from the nucleus accumbens and striatum, due to their key role in the brain’s reward circuitry [60,61]. However, recent studies also applied metabolomics on blood and urine samples. As disorders of the central and peripheral nervous systems might be reflected in changes of these more easily available specimens, applying metabolomics to urine or blood might serve as a valuable diagnostic tool for the assessment and treatment of addictive disorders [62].

Among substances studied, alcohol represents one of the substances with the highest public health impact due to the high prevalence and early onset of its use [72]. Alcoholism has even been considered to represent the most prevalent neuropsychiatric illness affecting modern societies today [63]. Metabolic profiling of rat brain revealed profound neurometabolomic alterations within brain circuits known to be highly sensitive to the long-term effects of alcohol (i.e., the infralimbic accumbens shell) [73]. The so far unrecognized pathophysiological mechanism of mitochondrial dysregulation in the accumbens shell has been suggested to represent an important mechanism involved in the pathophysiology of alcohol addiction [63]. A recent study conducted on mice treated with ethanol revealed perturbations in the fecal und urinary metabolome, indicating changes in essential pathways of energy generation, nitrogen and AA metabolism [64]. However, further studies are required to confirm whether or not these alterations might serve as a biomarker for assessing alcohol addiction.

Nicotine addiction represents a global health problem, as it affects one-third of the population [74]. The rewarding effects of nicotine are considered mainly responsible for its high addictive potential. To enable a deeper understanding of the underlying mechanisms of the effects of nicotine, endogenous brain metabolites have been investigated in nicotine-addicted mice. Alterations related to neurotransmitter, AA and energy metabolism as well as to the anti-oxidative stress response and membrane metabolism became evident [65].

Studies on animal brain have suggested that cocaine affects energy metabolism (i.e., inhibition of mitochondrial fatty acid oxidation), AA and biogenic amines metabolism, neurotransmitter (i.e., serotonin, glutamate, GABA) pathways, and pathways involved in oxidative stress response energy metabolism as summarized in Table 4. Additionally, changes in glucose metabolism, differing among anatomical areas, became evident [66]. One study also investigated the effect of cocaine addiction on the liver metabolome [67]. Besides toxicity on the central nervous system, cocaine-induced hepatoxicity has been linked to mortality in cocaine abusers [75]. Changes in lipid metabolism, i.e., inhibition of mitochondrial fatty acid oxidation, were observed, thereby contributing to the understanding of the pathogenesis of cocaine-induced fatty liver development. In a study conducted on plasma as well as urine samples of cocaine-addicted rats, changes observed in plasma were not accompanied by changes in the urine metabolome [62]. The authors argued that a longer sampling time for urine might have masked the effect of cocaine addiction. Hence, it is important to consider the possible effects of sampling times in future studies. Moreover, it has to be kept in mind that species-differences are common in psychiatric disorders. In this regard, the mouse has been shown to be more susceptible to cocaine-induced hepatotoxicity compared to the rat [76].

Mapping metabolic “signatures” to identify drug abuse, is of special interest in drugs that are rapidly metabolized, such as heroin, which has an approximate half-life of 2–4 min [77]. In a study conducted on serum and urine of rats administered heroin for a prolonged time period, Zheng et al. [69] identified tryptophan and 5-hydroxytryptamine in serum, as well as tryptophan and 5-hydroxyindolacetate in urine, as potential biomarkers of long-term heroin addiction.

Although opioid analgesics represent the gold standard for pain management, they show serious side effects of morphine, such as addiction, immunosuppression and gastrointestinal symptoms [78]. Studies in morphine-addicted rats indicate that next to changes in AA and biogenic amine-metabolism, disruption of energy metabolism is of especially high relevance in morphine addiction, as alterations in urine TCA-cycle metabolites were observed in rats [62]. In recent years, growing research interest emphasized on the interactions between the central nervous system and the gut microbiome have revealed a potential contribution of gut dysbiosis in the pathogenesis of addictive disorders [79]. Wang et al. [78] explored the gut metabolomic profile of morphine-addicted mice, to identify functional changes in the gut microbiome, as gut metabolites represent an important link between the gastrointestinal microbiome and the biological functions of the host. Morphine administration caused changes in the microbial and metabolomic profile, being indicative of dysbiosis and expansion of potential pathogens, which might contribute to the deleterious effects of opioid use. More specifically, impairment in bile acids and morphine-2-glucuronide/morphine biotransformation as well as alterations in deoxycholic acid and phosphatidylethanolamines were observed. These changes went along with increases in bacterial communities associated with pathogenic functions and a decrease in those associated with stress tolerance, such as an expansion of Enterococcus faecalis, a well-known pathogen in humans [80].

The effect of methamphetamine addiction on the metabolic profiles of the brain, plasma, and urine in rodents was assessed as depicted in Table 4. While Shima et al. [71] identified mainly metabolites related to energy metabolism as potential biomarkers for acute toxicity of methamphetamine (e.g., lower concentration of the ketone body 3-hydroxybutyrate in plasma as well as urine), Zaitsu et al. [62] observed no changes in metabolites related to energy metabolism. Therefore, different biological states in methamphetamine addiction vs. methamphetamine acute intoxication have to be considered.

Overall, studies presented on drug addiction models demonstrate that the choice of appropriate sampling material plays an important role in the study of underlying mechanisms causing addictive behavior. As blood and/or urine metabolic profiles cannot measure the direct contribution to the central nervous system or peripheral regions, further studies in humans conducted on cerebrospinal fluid should be conducted to describe the biological states in drug addiction.

## 3. Examples of Using Metabolomics to Test Treatment Strategies

Pharmacometabolomic studies using animal models have been conducted to investigate the effect of the treatment of several mental disorders. The following sections provide examples of these studies among antidepressants, mood stabilizers, anxiolytics, and antipsychotics.

### 3.1. Antidepressants

In patients suffering from MDD, response rates to antidepressants are low, duration to attain therapeutic benefit is long and many patients drop out of care [18]. Thus, biomarkers to identify subgroups of patients and to offer precisely targeted treatment are needed to maximize the likelihood of treatment response or remission, while simultaneously minimizing the detrimental side effects [81,82].

As metabolomics helps to reveal the underlying biochemical processes of MDD, it holds potential to study the effects of treatment on those biological processes. This is of special interest in MDD resistant to current medications, where metabolomics might offer a method to evaluate the likelihood of a successful response [3]. In this regard, animal models of depression represent tools to elucidate the mechanism of antidepressant agents to reveal their efficacy in the treatment of MDD. Due to the heterogeneity of depressive symptoms and various co-occurring disorders, drug discovery and development for MDD represents several challenges. As antidepressants represent the most commonly prescribed drugs in developed countries, such as the USA, understanding the action of drug candidates is of utmost importance to evaluate their efficacy as well as safety [1]. Metabolomics might enable the comprehensive characterization of drug-induced metabolic changes in biological systems.

Previous studies in humans looked at a wide array of blood metabolites including lipids, AA, hormones, and biogenic amines to predict patient response to drug therapy [3]. More specifically, metabolomics has been applied following treatments with specific antidepressants, including sertraline [83], escitalopram [84], ketamine, and esketamine [84], as well as combinations of antidepressants (bupropion-escitalopram combination, venlafaxine-mirtazapine combination [85]). However, despite these studies already conducted in humans, animal models offer further important insights into the study of the therapeutic actions of drugs, thereby facilitating the development of novel treatments. Several previous studies investigated how antidepressants regulate molecular and neurobiological changes in functional brain regions. Metabolomics is considered as a powerful tool to gain insight into the molecular mechanisms targeted by pharmacotherapies [86].

Pharmacometabolomic studies using animal models have been used to investigate the effects of chronic treatment with antidepressants, as the long-term effects of most antidepressants remain unclear. However, as these medicaments typically have side effects, patients given antidepressants often show high levels of discontinuation [87]. Thus, revealing the underlying mechanisms of antidepressants is required to optimize the therapy of MDD and develop novel therapeutic drugs. Among antidepressants, venlafaxine represents one of the most prescribed drugs [88]. Metabolomic analysis of the hippocampus and prefrontal cortex in rats revealed significant alterations of the metabolic profiles of both brain areas, as well as correlations of the metabolic profile of the hippocampus with depression-related despair behavior [86]. More specifically, venlafaxine upregulated most metabolites in the analyzed brain regions, suggesting an enhanced metabolic capacity due to antidepressant treatment. The altered metabolites had typical regional specificity, suggesting that different mechanisms in the two brain regions were responsible for the antidepressant effect. Lower levels of glycine and methyl phosphate, as well as higher levels of arachidonic acid, N-acetyltryptophan, β-alanine, oleic acid and oxalic acid, were observed in the hippocampus. While metabolites of the two studied brain regions had different trends, 3-hydroxybutyric acid was upregulated in both brain regions. Other overlapping metabolites were identified, whereby they were involved in the functions of cell death and survival, lipid metabolism and small molecule biochemistry.

In addition to this, studies on ketamine treatment in humans are complemented by animal studies. A recent study conducted in mice treated with ketamine was conducted to reveal its effects on the hippocampus [89]. Compared to a control group, several metabolites related to AA, energy metabolism and oxidative stress were identified. Furthermore, the potential of a prophylactic ketamine treatment was investigated in mice exposed to a stressful experience [90]. Changes in metabolites related to tryptophan metabolism were observed, which corroborated changes measured in human plasma due to prophylactic ketamine treatment [91]. Overall, prophylactic ketamine treatment attenuated learned fear and changed metabolites in the prefrontal cortex, hippocampus, and plasma, mainly those involved in purine and pyrimidine metabolism as well as neurotransmission. More specifically, precursors to inhibitory neurotransmitters were elevated, whereas the opposite was observed for excitatory neurotransmitters. Thus, first insights into the biological processes occurring in the brain and plasma that are critical to maintaining a long-lasting resilience against stress-induced disorders were provided by this animal model study.

In cynomolgus macaques, ketamine injection affected major energy and AA pathways detected in urine and serum [28]. Ketamine caused a decrease in serum lactate, α-glucose, and, myo-inositol, as well as lower levels of lactate, pyruvate, and succinate in urine.

As conventional antidepressants lack efficacy for many patients, there is a high demand for new effective antidepressants. Among novel antidepressants, Chinese medicine has been suggested to provide effective ways in the discovery of new antidepressants. Recently, the effect of a secoiridoid compound used in Chinese herbal medicine (gentiopicroside) as an antidepressant was investigated in a corticosterone-induced depression model in rats using metabolomic analyses of the hippocampus [92]. The study revealed a protecting effect of gentiopicroside on corticosterone-induced apoptosis and oxidative stress on brain cells by suppressing arachidonic acid levels. Differently expressed metabolites correlated with metabolic pathways involved in homocysteine degradation, sphingolipid, AA, and α-linolenic acid metabolism.

A further key constituent of many traditional Chinese medicines is chlorogenic acid, a phytogenic herbal extract, with claimed antidepressant and neuroprotective effects [93]. In a study using a rat model of tricyclic antidepressant treatment-resistant depression, the antidepressant-like effects of chlorogenic acids on the urinary metabolome were investigated. Perturbations in several urinary metabolites related to energy metabolism, neurotransmitter metabolism and AA metabolism (such as 5-hydroxytryptamine, adrenocorticotropic hormone, corticotropin-releasing hormone, dopamine, alanine, propanedioic acid, threonine, serine, myo-inositol, acetic acid, creatinine, hippurate, ferulic acid, kynurenic acid) in depressed compared to normal rats were reversed with chlorogenic acid [94].

Scopolamine, a drug typically used in the treatment of nausea and motion sickness has been investigated as a potential rapidly acting antidepressant for MDD. In a study conducted in a mice model of treatment-resistant depression, the effects of scopolamine and ketamine on metabolomic analysis of the frontal cortex and brain were evaluated [95]. Results suggest that α-amino-3-hydroxy-5-methyl-4-isoxazole propionic acid (AMPA) and mammalian target of rapamycin (mTOR) signaling pathways are important for the antidepressant effects and of high importance for the treatment of resistant depression.

To sum up, based on the examples provided above, animal models of depression provide a basis for evaluating the potential efficacy as well as long-term detrimental side effects of drugs for the clinical treatment of depression.

### 3.2. Mood Stabilizers

Bipolar Disorder (BD) is a common, severe and persistent mental disorder, characterized by alternating periods of extreme mood states of depression and mania [96]. In contrast to MDD, fewer studies were carried out on this disorder, contributing to the so far poor knowledge of its pathophysiology [2]. Additionally, the mechanisms of actions of therapies remain largely unknown. Although animal models can be useful in understanding pathological mechanisms associated with psychiatric illnesses, there is currently a lack of adequate animal models for bipolar disorders. While animal models have been created to mimic depression or mania, only a few have been created that include the cyclical nature of the human condition [97]. To our best knowledge, until now no metabolomics study have been conducted on animal models investigating the underlying nature of this disorder, whereas one study aimed to elucidate the effect of mood stabilizers. More specifically, changes in metabolites related to glutamate and energy metabolism in brain tissue following the treatment with valproate or lithium [96], the mainstays of BD treatment [98], were observed. In that study, these changes were compared to postmortem brain analysis in BD patients. Several metabolites were inversely altered in treated rats compared to patients who died with a diagnosis of BP. While glutamate levels were increased in the human post-mortem brain, the ratio of glutamate to glutamine was decreased due to the valproate treatment and GABA was enhanced following lithium treatment. Thus, this study suggests that the equilibrium of excitatory and inhibitory neurotransmission is of particular relevance in BD [96]. Additionally, creatine and myo-inositol were concordantly altered in both the animal and the human tissues. Overall, this study provides some indications that a reversal of metabolic disturbances in the brain may be crucial for a mood-stabilizing effect. However, one has to consider that the postmortem tissue samples used in that study derived from patients both with and without a medication history and medication treatment history of the patients was not controlled for [96]. Despite this limitation, this study provides an example of how data obtained from animal models can be linked to human data, to identify key metabolites associated with both the pathophysiology as well as the treatment of psychiatric disorders.

### 3.3. Anxiolytics

A study conducted on rats investigated the effect of the treatment with alprazolam, a drug known for its anxiolytic effects. Decreased urine concentrations of succinic acid were observed, which was related to mitochondrial tricarboxylic acid cycle (TCA) energy generation [99].

### 3.4. Antipsychotics

In a study of antipsychotics in humans, ideally, drug-naive individuals should be examined, to avoid confounding effects of varying treatment options. However, studies on postmortem brain tissue in humans are typically conducted on individuals who have been treated with varying lifetime antipsychotic medication doses [48,49]. The central treatment of schizophrenia relies on antipsychotic drugs, which can lead to severe, and sometimes irreversible side effects, especially extrapyramidal symptoms [100]. For the development of drugs with a lower risk for such debilitating side effects, the knowledge of the underlying mechanisms that cause side effects is a prerequisite. To study the biological mechanisms of antipsychotic drugs on these side effects, drug-naive animals represent a valuable model. Animal models of antipsychotic drug treatment [101,102] recognized changes in energy (i.e., glucose), lipid (i.e., sphingolipids), protein (i.e., leucine) and neurotransmitter (i.e., glutamate and aspartate) metabolism, with a generally high heterogenicity among antipsychotic drugs. The concept that aberrations in the level of glutamatergic neurotransmission and myelin synthesis significantly contribute to schizophrenia-as revealed by animal studies—is supported by postmortem analysis of human brains [103]. However, the drawback of profiling studies in human postmortem brain compared to animal studies is, that they are commonly applied to individuals that have been typically exposed to various antipsychotic medications [48,49]. Based on chronic antipsychotic treatment in drug-naive rodents it was assumed that demyelination plays a major role in the pathophysiology of extrapyramidal symptoms [101]. These results provide a basis for the identification of novel drugs that show a lower risk of unwanted side-effects.

## 4. Strengths and Limitations of Animal Models

As described above, samples from the most relevant biological material for identifying biomarkers of psychiatric diseases derived from the brain, which are typically only available postmortem from humans. Thus, animal models are essential to study causal links between a detected psychiatric pathology and affected molecular pathways [3].

A further main aspect highlighting the importance of animal models is that various factors, such as sex, age, nutritional status, environmental fluctuation, and circadian fluctuation readily affect metabolic profiles [6,104,105,106]. Thus, animal models are indispensable for the basic study of the effect of human mental disorders on metabolic profiles, as they enable the reduction in the variability of external factors. However, one has to keep in mind that animals do not share most of those factors (i.e., lifestyle and environmental factors) with humans. Thus, observed underlying biochemical mechanisms of psychiatric disorders might not be shared in animals and humans.

Other benefits include the option to study the side effects of chronic drug administrations, such as the effect of chronic antipsychotic treatment on the central nervous system. A deeper understanding of the underlying biological mechanisms could improve the development of novel drugs with reduced unwanted side-effects [101].

Animal models are not only required for the direct study of brain metabolites but also in the study of the gut microbiome. While the human microbiome is the product of complex environmental interactions and highly diverse [107], germ-free animals that are raised in sterile environments enable the investigation of the effects of the gut microbiome on behavior [79,108]. Animals lacking any internal or external microbiome can be colonized with microbial communities from donor animals or humans, to elucidate the contribution of a defined microbiome to a phenotype of interest [108]. However, studies in germ-free animals also have several limitations, such as the high technical requirements and costs. Furthermore, the gut microbiome significantly affects the development of the gastrointestinal tract and immune systems, thereby complicating assessments in adult animals [109,110]. Thus, studies in germ-free animals offer a valuable option to subsequently assess mechanistic contributions of specific microbial strains [79].

However, there is no question that many assumptions are intrinsic to animal models of human diseases. For instance, there are several anatomical, physiological and environmental differences between humans and animals. A further complication is the high heterogeneity of most psychiatric disorders, likely due to high variance in the causal factors despite a similar phenotype [111]. Moreover, animals will never fully re-capitulate the broad criteria for many mental illnesses, including psychiatric symptoms that are uniquely human such as delusions, suicidal thoughts or guilty ruminations [112]. Therefore, an animal model that reproduces a whole psychiatric syndrome, such as MDD, BD or schizophrenia, is almost impossible in a lower animal [20]. Thus, animal models only provide mechanistic knowledge in the exploration of mechanisms underlying biological processes that are relevant for psychiatric diseases. However, this knowledge is required to understand the full complexity of the brain–particularly how molecular and cellular processes of the brain drive behavior. Thus, carefully chosen animal studies provide a starting point in the development of novel interventions. Nevertheless, we have to keep in mind that no animal model for human disease does so, even for organic diseases, such as cardiovascular diseases [113].

In conclusion, despite the limited translational value to the clinical situation, animal models provide some direct insights into the cellular metabolites produced during psychiatric processes. Based on data derived from animal models, it is possible to go back to diagnostic and prognostic markers for psychiatric disorders, which so far lack a reliable and robust diagnosis and therapeutic options. Thus, a combination of representative animal models and human studies on cerebrospinal fluid, combined with metabolomic approaches might improve the insight into mental disorders and advance the development of novel treatment strategies. Moreover, the application of metabolomics *per se* also offers the option to bypass many of the assumptions of animal models by applying this approach to biofluids that are easily accessible, such as blood, urine and saliva.

## Figures and Tables

**Table 1:** Possible biomarkers identified in animal models of depression.

Subject	Sampling Material	Analytical Technique	Metabolites Identified	Pathways Involved/Functions	Reference
Rats	Brain (prefrontal cortex)	non-targeted GC-MS ^1^	GABA ^2^ glutamine, methionine, adenosine, proline, alanine, cysteamine, 1-methylhydantoin, creatine, myo-inositol, N-oleoyldopamine, trehalose-6-phosphate, phosphate, N-acetyl-L-leucine, acetylsalicyclic acid, N-acetyltryptophan, phosphomycin, glycylproline	AA ^3^ metabolism, lipid metabolism, glucose metabolism	[23]
Mice	Brain (prefrontal cortex)	targeted LC-MS/MS ^4^	Glutamate, L-DOPA, vanillylmandelic acid	GABAergic and catecholaminergic pathways	[1]
Rats	Brain (prefrontal cortex)	non-targeted GC-MS	Proline, creatine, taurine, glycerol, isoleucine, GABA, glutamate, glutamine, asparagine, N-acetyl aspartate, stearic acid, palmitic acid, uracil, β-alanine	AA metabolism, energy metabolism, lipid metabolism, disturbances in neurotransmitters	[24]
Rats	Brain (hippo-campus)	non-targeted GC-MS	Glutamate, glutamine, glycine, arachidonic acid, hexadecane, 2-monopalmitin, methyl palmitoleate, ethanolamine, o-phosphorylethanolamine	AA metabolism, lipid metabolism	[25]
Rats	Brain (cere-bellum)	non-targeted GC-MS	Glycine, adenosine, 3-hydroxybutyric acid, creatinine, 2,5-dihydroxypyrazine, pantothenic acid, dihydroxyacetone phosphate, proline, phenylalanine, tyrosine, lysine, glutamine	AA metabolism, energy metabolism	[26]
Macaques	(1) Cerebro-spinal fluid	non-targeted GC-MS	Glycine, threonine, acetic acid, propanoic acid, butanoic acid, oleic acid, octadecanoic acid, hexadecenoic acid, myo-inositol	AA metabolism, fatty acid biosynthesis, ABC transport system	[15]
	(2) Serum		Threonine, butanoic acid, serine, leucine, methionine, citric acid, leucine, myo-inositol	AA metabolism, fatty acid biosynthesis, ABC transport system	

^1^ GC-MS, gas chromatography–mass spectrometry. ^2^ GABA, γ-aminobutyric acid. ^3^ AA, amino acid. ^4^ LC-MS/MS, liquid chromatography–tandem mass spectrometry.

**Table 2:** Possible biomarkers identified in animal-based studies of anxiety.

Subject	Sampling Material	Analytical Technique	Metabolites Identified	Pathways Involved/Functions	Reference
Mice	(1) Brain (cingulate cortex)	targeted LC-MS/MS ^1^	1-methyl histidine, deoxyuridine, kynurenic acid, carnitine, acetylcarnitine	AA ^2^ metabolism, neurotransmitter metabolism, pyruvate metabolism, oxidative stress, apoptosis	[35]
	(2) Plasma		1-methyl histidine, deoxyuridine, kynurenic acid, 2-hydroxygluterate, cytosine	AA metabolism, neurotransmitter metabolism, pyruvate metabolism, oxidative stress, apoptosis	
Mice	Brain (cingulate cortex)	non-targeted GC-MS ^3^	Dehydroascorbate, xylose, succinic acid	Energy metabolism, mitochondrial import and transport, oxidative stress, neurotransmission	[36]
Dogs	Plasma	non-targeted LC-MS ^4^	Glutamine, γ-glutamyl-glutamine	Glutamine metabolism	[38]

^1^ LC-MS/MS, liquid chromatography- tandem mass spectrometry. ^2^ AA, amino acid. ^3^ GC-MS, gas chromatography–mass spectrometry. ^4^ LC-MS, liquid chromatography- mass spectrometry.

**Table 3:** Possible biomarkers identified in animal-based studies of schizophrenia.

Subject	Sampling Material	Analytical Technique	Metabolites Identified	Pathways Involved/Functions	Reference
Rats	Brain (cortex, hippo-campus)	^1^ H-MAS-NMR ^1^	Glutamate, glutamine, citrate, succinate, aspartate, alanine, acetate, L-serine	Glutamate synthesis, Krebs cycle, energy metabolism	[52]
Rats	Brain (prefrontal cortex)	LC-MS ^2^	L-tyrosine, γ-glutamylglutamine, L-citrulline, L-cysteine, 2-phenylacetamide, phenylpyruvate, 2,3-butanedione, cytosine, GABA ^3^, O-acetylcarnitine, adenylosuccinate, guanine, carnitine	Glutamate metabolism, glutamatergic neurotransmission, arginine and proline metabolism, purine reactions	[56]

^1^ H-MAS-NMR, proton magic angle spinning nuclear magnetic resonance. ^2^ LC-MS, liquid chromatography–mass spectrometry. ^3^ GABA, γ-aminobutyric acid.

**Table 4:** Possible biomarkers identified in animal-based studies of various addictive disorders.

Substance	Subject	Analytical Technique	Sampling Material	Metabolites Identified	Pathways Involved/Functions	Reference
Alcohol	Rats	non-targeted LC-MS ^1^	Brain (cortical: prelimbic and infralimbic; striatal: accumbens core and shell)	Dopamine, Met-enkephalin	Energy metabolism in the accumbens shell	[63]
	Rats	targeted LC-MS/MS ^2^	(1) Urine	cytosine, hypoxanthine, thiamine, uracil, uridine, acetylcarnitine, glutamine, alanine, aspartate, glycine, serine, threonine, valine, leucine, isoleucine, phenylalanine, tyrosine	Energy metabolism, Nitrogen metabolism, AA ^3^ metabolism	[64]
			(2) Feces	uracil, glutamine, glycine, leucine, putrescine, acetylcarnitine	Energy metabolism, Nitrogen metabolism, AA metabolism	
Nicotine	Mice	^1^ H-NMR ^4^	Brain (nucleus accumbens, striatum)	glutamate, tryptamine, acetylcholine, glucose, lactate, creatine, 3-hydroxybutyrate, nicotinamide-adenine dinucleotide (NAD), glutathione, taurine, phosphocholine, glycerol	Neurotransmitter disturbance, Energy metabolism, AA metabolism, membrane metabolism, dysregulation of anti-oxidative stress response	[65]
Cocaine	Rats	non-targeted GC-MS ^5^	(1) Plasma	Threonine, cystine, n-propylamine, spermidine	Stress response, immune response	[62]
			(2) Urine	No changes		
	Rat	IMMS ^6^	Brain (frontal cortex, striatum, thalamus)	Serotonin, norepinephrine, glucose, dopamine, DOPAC, 5-HIAA	Glucose metabolism, biogenic amine metabolism (esp. glycolysis metabolome in the thalamus)	[66]
	Mice	non-targeted LC-MS	Liver, serum	Long-chain acetylcarnitines (i.e., palmitoyl-carnitine), phospholipids	Lipid metabolism (inhibition of mitochondrial β-oxidation)	[67]
	Rat	^1^ H-NMR	Brain (hippo-campus, nucleus accumbens, prefrontal cortex, striatum)	Neurotransmitter (glutamate, GABA ^7^), creatine, taurine, n-acetylaspartate, lactate; choline, phosphocholine, glycerol, leucine, lycine, cysteine	Energy metabolism (mitochondrial dysregulation), membrane disruption, neurotransmitter disturbance, oxidative stress, AA metabolism	[68]
Heroin	Rats	non-targeted GC-MS	(1) Serum	Tryptophan, 5-hydroxytryptamine, leucin, aspartate, phenylalanine, hydroxyproline citrate, 9-hexadecenoic acid, palmitic acid	Energy metabolism (TCA-cycle ^8^, free fatty acid metabolism), lipid metabolism, AA turnover	[69]
			(2) Urine	Tryptophan, hepatanedioic acid, azelate, 5-hydroxyindoleacetat		
Morphine	Rats	non-targeted GC-MS	(1) Plasma	3-hydroxybutyric acid, tryptophan, cystine, n-propylamine	AA metabolism (tryptophan uptake from blood by brain), energy metabolism (reduced β-oxidation from fatty acids and/or ketone production form acetyl Co A)	[62]
			(2) Urine	2-ketoglutaric acid, fumaric acid, malic acid, threonine, glutamic acid, isoleucine, valine, aspartic acid, oxamic acid, 2-aminoethanol, indoxyl sulfate, creatinine,	Energy metabolism (via TCA-cycle disruption), neurotransmitter metabolism (disruption of biotransformation of glutamic acid to 2-ketoglutaric acid)	
Meth-amphet-amine	Mice	non-targeted GC-MS and non-targeted LC-MS	Brain (whole brain)	Homocarnisine, 4-guanidinobutanoate, pantothenate, myo-inositol	GABAergic metabolism	[70]
	Rat	targeted GC-MS and CE-MS/MS ^9^	(1) Plasma	Glucose, 3-hydroxybutyrate	Energy metabolism (oxidative phosphorylation via TCA-cycle, glycolysis), fatty acid metabolism	[71]
			(2) Urine	5-oxoproline, saccharic acid, uracil, 3-hydroxybutyrate, adipic acid, fumarate, α-ketoglutarate	TCA-cycle, fatty acid metabolism	
	Rats	non-targeted GC-MS	(1) Plasma	n-propylamine, lauric acid		[62]
			(2) Urine	lactose, spermidine, stearic acid		

^1^ LC-MS, liquid chromatography–mass spectrometry. ^2^ LC-MS/MS, liquid chromatography–tandem mass spectrometry. ^3^ AA, amino acid. ^4^ H-NMR, proton nuclear magnetic resonance. ^5^ GC-MS, gas chromatography–mass spectrometry. ^6^ IMMS, ion mobility mass spectrometry. ^7^ GABA, γ-aminobutyric acid. ^8^ TCA, tricarboxylic acid cycle. ^9^ CE-MS/MS, capillary electrophoresis–tandem mass spectrometry.

## References

[1] Wang W., Guo H., Zhang S.-X., Li J., Cheng K., Bai S.-J., Yang D.-Y., Wang H.-Y., Liang Z.-H., Liao L. (2016). Targeted Metabolomic Pathway Analysis and Validation Revealed Glutamatergic Disorder in the Prefrontal Cortex among the Chronic Social Defeat Stress Mice Model of Depression. J. Proteome Res..

[2] Sethi S., Brietzke E. (2016). Omics-Based Biomarkers: Application of Metabolomics in Neuropsychiatric Disorders. Int. J. Neruopsychopharmacol..

[3] Guest P.C., Guest F.L., Martins-de Souza D. (2016). Making Sense of Blood-Based Proteomics and Metabolomics in Psychiatric Research. Int. J. Neruopsychopharmacol..

[4] Cuthbert B.N., Insel T.R. (2013). Toward the future of psychiatric diagnosis: The seven pillars of RDoC. BMC Med..

[5] Atkinson A.J., Colburn W.A., DeGruttola V.G., DeMets D.L., Downing G.J., Spilker B.A., Biomarkers Definitions Working Group (2001). Biomarkers and surrogate endpoints: Preferred definitions and conceptual framework. Clin. Pharmacol. Ther..

[6] Dettmer K., Aronov P.A., Hammock B.D. (2007). Mass spectrometry-based metabolomics. Mass Spectrom. Rev..

[7] Kaddurah-Daouk R., Krishnan K.R.R. (2009). Metabolomics: A Global Biochemical Approach to the Study of Central Nervous System Diseases. Neuropsychopharmacology.

[8] Pasikanti K.K., Ho P.C., Chan E.C.Y. (2008). Gas chromatography/mass spectrometry in metabolic profiling of biological fluids. J. Chromatogr. B.

[9] Kaddurah-Daouk R., Kristal B.S., Weinshilboum R.M. (2008). Metabolomics: A Global Biochemical Approach to Drug Response and Disease. Annu. Rev. Pharmacol. Toxicol..

[10] Wood P.L. (2014). Mass Spectrometry Strategies for Clinical Metabolomics and Lipidomics in Psychiatry, Neurology, and Neuro-Oncology. Neuropsychopharmacology.

[11] Lozupone M., Seripa D., Stella E., La Montagna M., Solfrizzi V., Quaranta N., Veneziani F., Cester A., Sardone R., Bonfiglio C. (2017). Innovative biomarkers in psychiatric disorders: A major clinical challenge in psychiatry. Expert Rev. Proteom.

[12] Renoir T., Hasebe K., Gray L. (2013). Mind and body: How the health of the body impacts on neuropsychiatry. Front. Pharmacol..

[13] Nedic Erjavec G., Konjevod M., Nikolac Perkovic M., Svob Strac D., Tudor L., Barbas C., Grune T., Zarkovic N., Pivac N. (2018). Short overview on metabolomic approach and redox changes in psychiatric disorders. Redox Biol..

[14] Depression. https://www.who.int/news-room/fact-sheets/detail/depression.

[15] Deng F.-L., Pan J.-X., Zheng P., Xia J.-J., Yin B.-M., Liang W.-W., Li Y.-F., Wu J., Xu F., Wu Q.-Y. (2019). Metabonomics reveals peripheral and central short-chain fatty acid and amino acid dysfunction in a naturally occurring depressive model of macaques. Neuropsychiatr. Dis. Treat..

[16] Daly E.J., Trivedi M.H., Wisniewski S.R., Nierenberg A.A., Gaynes B.N., Warden D., Morris D.W., Luther J.F., Farabaugh A., Cook I. (2010). Health-related quality of life in depression: A STAR*D report. Ann. Clin. Psychiatry.

[17] Insel T., Cuthbert B., Garvey M., Heinssen R., Pine D.S., Quinn K., Sanislow C., Wang P. (2010). Research Domain Criteria (RDoC): Toward a New Classification Framework for Research on Mental Disorders. Am. J. Psychiatry.

[18] Gadad B.S., Jha M.K., Czysz A., Furman J.L., Mayes T.L., Emslie M.P., Trivedi M.H. (2018). Peripheral biomarkers of major depression and antidepressant treatment response: Current knowledge and future outlooks. J. Affect. Dis..

[19] Strimbu K., Tavel J.A. (2010). What are biomarkers?. Curr. Opin. HIV AIDS.

[20] Nestler E.J., Hyman S.E. (2010). Animal models of neuropsychiatric disorders. Nat. Neurosci..

[21] Willner P. (2005). Chronic Mild Stress (CMS) Revisited: Consistency and behavioural-neurobiological concordance in the effects of CMS. Neuropsychobiology..

[22] Liu L.-Y., Zhang H.-J., Luo L.-Y., Pu J.-B., Liang W.-Q., Zhu C.-Q., Li Y.-P., Wang P.-R., Zhang Y.-Y., Yang C.-Y. (2018). Blood and urinary metabolomic evidence validating traditional Chinese medicine diagnostic classification of major depressive disorder. Chin. Med..

[23] Zhou X., Liu L., Zhang Y., Pu J., Yang L., Zhou C., Yuan S., Zhang H., Xie P. (2017). Metabolomics identifies perturbations in amino acid metabolism in the prefrontal cortex of the learned helplessness rat model of depression. Neuroscience.

[24] Liu L., Zhou X., Zhang Y., Liu Y., Yang L., Pu J., Zhu D., Zhou C., Xie P. (2016). The identification of metabolic disturbances in the prefrontal cortex of the chronic restraint stress rat model of depression. Behav. Brain Res..

[25] Zhang Y., Yuan S., Pu J., Yang L., Zhou X., Liu L., Jiang X., Zhang H., Teng T., Tian L. (2018). Integrated Metabolomics and Proteomics Analysis of Hippocampus in a Rat Model of Depression. Neuroscience.

[26] Shao W., Chen J., Fan S., Lei Y., Xu H., Zhou J., Cheng P., Yang Y., Rao C., Wu B. (2015). Combined Metabolomics and Proteomics Analysis of Major Depression in an Animal Model: Perturbed Energy Metabolism in the Chronic Mild Stressed Rat Cerebellum. Omics J. Integr. Biol..

[27] Liu Y., Yieh L., Yang T., Drinkenburg W., Peeters P., Steckler T., Narayan V.A., Wittenberg G., Ye J. (2016). Metabolomic biosignature differentiates melancholic depressive patients from healthy controls. BMC Genom..

[28] Pan X., Zeng X., Hong J., Yuan C., Cui L., Ma J., Chang Y., Hua X. (2016). Effects of Ketamine on Metabolomics of Serum and Urine in Cynomolgus Macaques (Macaca fascicularis). J. Am. Assoc. Lab. Anim. Sci..

[29] Kaddurah-Daouk R., Yuan P., Boyle S.H., Matson W., Wang Z., Zeng Z.B., Zhu H., Dougherty G.G., Yao J.K., Chen G. (2012). Cerebrospinal Fluid Metabolome in Mood Disorders-Remission State has a Unique Metabolic Profile. Sci. Rep..

[30] Lochhead J.J., Ronaldson P.T., Davis T.P. (2017). Hypoxic Stress and Inflammatory Pain Disrupt Blood-Brain Barrier Tight Junctions: Implications for Drug Delivery to the Central Nervous System. AAPS J..

[31] Powell T.R., Tansey K.E., Breen G., Farmer A.E., Craig I.W., Uher R., McGuffin P., D’Souza U.M., Schalkwyk L.C. (2013). ATP-binding cassette sub-family F member 1 (ABCF1) is identified as a putative therapeutic target of escitalopram in the inflammatory cytokine pathway. J. Psychopharmacol..

[32] Huang X., Yu T., Li X., Cao Y., Li X., Liu B., Yang F., Li W., Zhao X., Feng G. (2013). *ABCB6*, *ABCB1* and *ABCG1* genetic polymorphisms and antidepressant response of SSRIs in Chinese depressive patients. Pharmacogenomics.

[33] Yu M., Jia H., Zhou C., Yang Y., Zhao Y., Yang M., Zou Z. (2017). Variations in gut microbiota and fecal metabolic phenotype associated with depression by 16S rRNA gene sequencing and LC/MS-based metabolomics. J. Pharma. Biomed. Anal..

[34] Roca M., Gili M., Garcia-Garcia M., Salva J., Vives M., Garcia Campayo J., Comas A. (2009). Prevalence and comorbidity of common mental disorders in primary care. J. Affect. Dis..

[35] Filiou M.D., Asara J.M., Nussbaumer M., Teplytska L., Landgraf R., Turck C.W. (2014). Behavioral extremes of trait anxiety in mice are characterized by distinct metabolic profiles. J. Psychiatr. Res..

[36] Filiou M.D., Zhang Y., Teplytska L., Reckow S., Gormanns P., Maccarrone G., Frank E., Kessler M.S., Hambsch B., Nussbaumer M. (2011). Proteomics and Metabolomics Analysis of a Trait Anxiety Mouse Model Reveals Divergent Mitochondrial Pathways. Biol. Psychiatry.

[37] Landgraf R., Kessler M.S., Bunck M., Spengler D., Zimbelmann M., Nussbaumer M., Czibere L., Turck C.W., Singewald N., Rujescu D. (2007). Candidate genes of anxiety-related behavior in HAB/LAB rats and mice: Focus on vasopressin and glyoxalase-I. Neurosci. Biobehav. Res..

[38] Puurunen J., Tiira K., Vapalahti K., Lehtonen M., Hanhineva K., Lohi H. (2018). Fearful dogs have increased plasma glutamine and γ-glutamyl glutamine. Sci. Rep..

[39] Schousboe A. (2019). Metabolic signaling in the brain and the role of astrocytes in control of glutamate and GABA neurotransmission. Neurosci. Lett..

[40] Matés J. (2002). Glutamine and its relationship with intracellular redox status, oxidative stress and cell proliferation/death. Int. J. Biochem. Cell Biol..

[41] Curi R., Lagranha C.J., Doi S.Q., Sellitti D.F., Procopio J., Pithon-Curi T.C., Corless M., Newsholme P. (2005). Molecular mechanisms of glutamine action. J. Cell. Physiol..

[42] Weiner I.D., Verlander J.W. (2019). Emerging Features of Ammonia Metabolism and Transport in Acid-Base Balance. Semin. Nephrol..

[43] Bergink V. (2004). Glutamate and anxiety. Eur. Neuropsychopharmacol..

[44] Cortese B.M., Phan K.L. (2005). The Role of Glutamate in Anxiety and Related Disorders. CNS Spectr..

[45] Riaza Bermudo-Soriano C., Perez-Rodriguez M.M., Vaquero-Lorenzo C., Baca-Garcia E. (2012). New perspectives in glutamate and anxiety. Pharmacol. Biochem. Behav..

[46] Bray N.J., Leweke F.M., Kapur S., Meyer-Lindenberg A. (2010). The neurobiology of schizophrenia: New leads and avenues for treatment. Curr. Opin. Neurobiol..

[47] van Os J., Kapur S. (2009). Schizophrenia. Lancet.

[48] Chan M.K., Tsang T.M., Harris L.W., Guest P.C., Holmes E., Bahn S. (2011). Evidence for disease and antipsychotic medication effects in post-mortem brain from schizophrenia patients. Mol. Psychiatry.

[49] Halim N.D., Lipska B.K., Hyde T.M., Deep-Soboslay A., Saylor E.M., Herman M.M., Thakar J., Verma A., Kleinman J.E. (2008). Increased lactate levels and reduced pH in postmortem brains of schizophrenics: Medication confounds. J. Neurosci. Method.

[50] Bubeníková-Valešová V., Horáček J., Vrajová M., Höschl C. (2008). Models of schizophrenia in humans and animals based on inhibition of NMDA receptors. Neurosci. Biobehav. Rev..

[51] Rung J.P., Carlsson A., Rydén Markinhuhta K., Carlsson M.L. (2005). (+)-MK-801 induced social withdrawal in rats; a model for negative symptoms of schizophrenia. Prog. Neuro-Psychopharmacol. Biol. Psychiatry.

[52] Sun L., Li J., Zhou K., Zhang M., Yang J., Li Y., Ji B., Zhang Z., Zhu H., Yang L. (2013). Metabolomic Analysis Reveals Metabolic Disturbance in the Cortex and Hippocampus of Subchronic MK-801 Treated Rats. PLoS ONE.

[53] Martins-de-Souza D., Harris L.W., Guest P.C., Bahn S. (2011). The Role of Energy Metabolism Dysfunction and Oxidative Stress in Schizophrenia Revealed by Proteomics. Antioxid. Redox Signal..

[54] Moghaddam B., Javitt D. (2012). From Revolution to Evolution: The Glutamate Hypothesis of Schizophrenia and its Implication for Treatment. Neuropsychopharmacology.

[55] Parsons C.G., Danysz W., Quack G. (1998). Glutamate in CNS disorders as a target for drug development: An update. Drug News Perspect..

[56] Xiao X., Dawson N., MacIntyre L., Morris B.J., Pratt J.A., Watson D.G., Higham D.J. (2011). Exploring metabolic pathway disruption in the subchronic phencyclidine model of schizophrenia with the Generalized Singular Value Decomposition. BMC Syst. Biol..

[57] Grant B.F., Goldstein R.B., Saha T.D., Chou S.P., Jung J., Zhang H., Pickering R.P., Ruan W.J., Smith S.M., Huang B. (2015). Epidemiology of *DSM.-5* Alcohol Use Disorder: Results From the National Epidemiologic Survey on Alcohol and Related Conditions III. JAMA Psychiatry.

[58] Grant B.F., Saha T.D., Ruan W.J., Goldstein R.B., Chou S.P., Jung J., Zhang H., Smith S.M., Pickering R.P., Huang B. (2016). Epidemiology of *DSM.-5* Drug Use Disorder: Results From the National Epidemiologic Survey on Alcohol and Related Conditions–III. JAMA Psychiatry.

[59] Spanagel R. (2017). Animal models of addiction. Dialog. Clin. Neurosci..

[60] Dalley J.W., Fryer T.D., Brichard L., Robinson E.S.J., Theobald D.E.H., Laane K., Pena Y., Murphy E.R., Shah Y., Probst K. (2007). Nucleus Accumbens D2/3 Receptors Predict Trait Impulsivity and Cocaine Reinforcement. Science.

[61] Maze I., Covington H.E., Dietz D.M., LaPlant Q., Renthal W., Russo S.J., Mechanic M., Mouzon E., Neve R.L., Haggarty S.J. (2010). Essential Role of the Histone Methyltransferase G9a in Cocaine-Induced Plasticity. Science.

[62] Zaitsu K., Miyawaki I., Bando K., Horie H., Shima N., Katagi M., Tatsuno M., Bamba T., Sato T., Ishii A. (2014). Metabolic profiling of urine and blood plasma in rat models of drug addiction on the basis of morphine, methamphetamine, and cocaine-induced conditioned place preference. Anal. Bioanal. Chem..

[63] Meinhardt M.W., Sévin D.C., Klee M.L., Dieter S., Sauer U., Sommer W.H. (2015). The Neurometabolic Fingerprint of Excessive Alcohol Drinking. Neuropsychopharmacology.

[64] Deda O., Virgiliou C., Orfanidis A., Gika H.G. (2019). Study of Fecal and Urinary Metabolite Perturbations Induced by Chronic Ethanol Treatment in Mice by UHPLC-MS/MS Targeted Profiling. Metabolites.

[65] Li H., Chen B., Shao X., Hu Z., Deng Y., Zhu R., Li Y., Zhang B., Hou J., Du C. (2014). 1H-Nuclear magnetic resonance-based metabolomic analysis of brain in mice with nicotine treatment. BMC Neurosci..

[66] Kaplan K.A., Chiu V.M., Lukus P.A., Zhang X., Siems W.F., Schenk J.O., Hill H.H. (2013). Neuronal metabolomics by ion mobility mass spectrometry: Cocaine effects on glucose and selected biogenic amine metabolites in the frontal cortex, striatum, and thalamus of the rat. Anal. Bioanal. Chem..

[67] Shi X., Yao D., Gosnell B.A., Chen C. (2012). Lipidomic profiling reveals protective function of fatty acid oxidation in cocaine-induced hepatotoxicity. J. Lipid Res..

[68] Li Y., Yan G.-Y., Zhou J.-Q., Bu Q., Deng P.-C., Yang Y.-Z., Lv L., Deng Y., Zhao J.-X., Shao X. (2012). 1H NMR-based metabonomics in brain nucleus accumbens and striatum following repeated cocaine treatment in rats. Neuroscience.

[69] Zheng T., Liu L., Aa J., Wang G., Cao B., Li M., Shi J., Wang X., Zhao C., Gu R. (2013). Metabolic phenotype of rats exposed to heroin and potential markers of heroin abuse. Drug Alcohol Depend..

[70] Adkins D.E., McClay J.L., Vunck S.A., Batman A.M., Vann R.E., Clark S.L., Souza R.P., Crowley J.J., Sullivan P.F., van den Oord E.J.C.G. (2013). Behavioral metabolomics analysis identifies novel neurochemical signatures in methamphetamine sensitization: Methamphetamine sensitization metabolomics. Genes Brain Behav..

[71] Shima N., Miyawaki I., Bando K., Horie H., Zaitsu K., Katagi M., Bamba T., Tsuchihashi H., Fukusaki E. (2011). Influences of methamphetamine-induced acute intoxication on urinary and plasma metabolic profiles in the rat. Toxicology.

[72] Shmulewitz D., Greene E.R., Hasin D. (2015). Commonalities and Differences across Substance Use Disorders: Phenomenological and Epidemiological Aspects. Alcohol Clin. Exp. Res..

[73] Meinhardt M.W., Hansson A.C., Perreau-Lenz S., Bauder-Wenz C., Stahlin O., Heilig M., Harper C., Drescher K.U., Spanagel R., Sommer W.H. (2013). Rescue of Infralimbic mGluR2 Deficit Restores Control Over Drug-Seeking Behavior in Alcohol Dependence. J. Neurosci..

[74] DiFranza J.R. (2008). Hooked from the First Cigarette. Sci. Am..

[75] Wanless I.R., Dore S., Gopinath N., Tan J., Cameron R., Heathcote E.J., Blendis L.M., Levy G. (1990). Histopathology of cocaine hepatotoxicity. Gastroenterology.

[76] Yao D., Shi X., Wang L., Gosnell B.A., Chen C. (2013). Characterization of Differential Cocaine Metabolism in Mouse and Rat through Metabolomics-Guided Metabolite Profiling. Drug Metab. Dispos..

[77] Dinis-Oliveira R.J. (2014). Metabolomics of drugs of abuse: A more realistic view of the toxicological complexity. Bioanalysis.

[78] Wang F., Meng J., Zhang L., Johnson T., Chen C., Roy S. (2018). Morphine induces changes in the gut microbiome and metabolome in a morphine dependence model. Sci. Rep..

[79] Meckel K.R., Kiraly D.D. (2019). A potential role for the gut microbiome in substance use disorders. Psychopharmacology.

[80] Fiore E., Van Tyne D., Gilmore M.S., Fischetti N., Ferretti P., Braunstein R. (2019). Pathogenicity of Enterococci. Gram-Positive Pathogens.

[81] Kessler R.C., Berglund P., Demler O., Jin R., Koretz D., Merikangas K.R., Rush A.J., Walters E.E., Wang P.S. (2003). The Epidemiology of Major Depressive Disorder: Results from the National Comorbidity Survey Replication (NCS-R). JAMA.

[82] Murray C.J.L. (2013). The State of US Health, 1990–2010: Burden of Diseases, Injuries, and Risk Factors. JAMA.

[83] Gupta M., Neavin D., Liu D., Biernacka J., Hall-Flavin D., Bobo W.V., Frye M.A., Skime M., Jenkins G.D., Batzler A. (2016). TSPAN5, ERICH3 and selective serotonin reuptake inhibitors in major depressive disorder: Pharmacometabolomics-informed pharmacogenomics. Mol. Psychiatry.

[84] Villaseñor A., Ramamoorthy A., Silva dos Santos M., Lorenzo M.P., Laje G., Zarate C., Barbas C., Wainer I.W. (2014). A pilot study of plasma metabolomic patterns from patients treated with ketamine for bipolar depression: Evidence for a response-related difference in mitochondrial networks: Metabolomics of ketamine response in depression. Br. J. Pharmacol..

[85] Czysz A.H., South C., Gadad B.S., Arning E., Soyombo A., Bottiglieri T., Trivedi M.H. (2019). Can targeted metabolomics predict depression recovery? Results from the CO-MED trial. Transl. Psychiatry.

[86] Bai S., Hu Q., Chen Z., Liang Z., Wang W., Shen P., Wang T., Wang H., Xie P. (2017). Brain region-specific metabolite networks regulate antidepressant effects of venlafaxine. RSC Adv..

[87] Demyttenaere K., Haddad P. (2000). Compliance with antidepressant therapy and antidepressant discontinuation symptoms. Acta Psychiatr. Scand..

[88] Lee K.-U., Lee Y.M., Nam J.-M., Lee H.-K., Kweon Y.-S., Lee C.T., Jun T.-Y. (2010). Antidepressant-Induced Sexual Dysfunction among Newer Antidepressants in a Naturalistic Setting. Psychiatry Investig..

[89] Lian B., Xia J., Yang X., Zhou C., Gong X., Gui S., Mao Q., Wang L., Li P., Huang C. (2018). Mechanisms of ketamine on mice hippocampi shown by gas chromatography–mass spectrometry-based metabolomic analysis. Neuroreport.

[90] McGowan J.C., Hill C., Mastrodonato A., LaGamma C.T., Kitayev A., Brachman R.A., Narain N.R., Kiebish M.A., Denny C.A. (2018). Prophylactic ketamine alters nucleotide and neurotransmitter metabolism in brain and plasma following stress. Neuropsychopharmacology.

[91] Rotroff D.M., Corum D.G., Motsinger-Reif A., Fiehn O., Bottrel N., Drevets W.C., Singh J., Salvadore G., Kaddurah-Daouk R. (2016). Metabolomic signatures of drug response phenotypes for ketamine and esketamine in subjects with refractory major depressive disorder: New mechanistic insights for rapid acting antidepressants. Transl. Psychiatry.

[92] Liu X., Liu C., Tian J., Gao X., Li K., Du G., Qin X. (2020). Plasma metabolomics of depressed patients and treatment with Xiaoyaosan based on mass spectrometry technique. J. Ethnopharmacol..

[93] Wu J., Chen H., Li H., Tang Y., Yang L., Cao S., Qin D. (2016). Antidepressant Potential of Chlorogenic Acid-Enriched Extract from Eucommia ulmoides Oliver Bark with Neuron Protection and Promotion of Serotonin Release through Enhancing Synapsin I Expression. Molecules.

[94] Zhao L., Zhang Z., Zhou M., Gou X., Zeng Y., Song J., Ma W., Xu Y. (2018). A urinary metabolomics (GC-MS) strategy to evaluate the antidepressant-like effect of chlorogenic acid in adrenocorticotropic hormone-treated rats. RSC Adv..

[95] Martin A.E., Schober D.A., Nikolayev A., Tolstikov V.V., Anderson W.H., Higgs R.E., Kuo M.-S., Laksmanan A., Catlow J.T., Li X. (2017). Further Evaluation of Mechanisms Associated with the Antidepressantlike Signature of Scopolamine in Mice. CNS Neurol. Disord. Drug Targets.

[96] Lan M.J., McLoughlin G.A., Griffin J.L., Tsang T.M., Huang J.T.J., Yuan P., Manji H., Holmes E., Bahn S. (2009). Metabonomic analysis identifies molecular changes associated with the pathophysiology and drug treatment of bipolar disorder. Mol. Psychiatry.

[97] Beyer K.E., Freund N. (2017). Animal models for bipolar disorder: From bedside to the cage. Int. J. Bipol. Dis..

[98] Belmaker R.H. (2004). Bipolar Disorder. N. Engl. J. Med..

[99] Sun F., Jiang Y., Yu Z., Zhang Z., Hu L., Cao G. (2018). Urine Metabolomics in Rats Treated by Alprazolam. Lat. Am. J. Pharm..

[100] Leucht S., Cipriani A., Spineli L., Mavridis D., Örey D., Richter F., Samara M., Barbui C., Engel R.R., Geddes J.R. (2013). Comparative efficacy and tolerability of 15 antipsychotic drugs in schizophrenia: A multiple-treatments meta-analysis. Lancet.

[101] McClay J.L., Vunck S.A., Batman A.M., Crowley J.J., Vann R.E., Beardsley P.M., van den Oord E.J. (2015). Neurochemical Metabolomics Reveals Disruption to Sphingolipid Metabolism Following Chronic Haloperidol Administration. J. Neuroimmune Pharmacol..

[102] McLoughlin G.A., Ma D., Tsang T.M., Jones D.N.C., Cilia J., Hill M.D., Robbins M.J., Benzel I.M., Maycox P.R., Holmes E. (2009). Analyzing the Effects of Psychotropic Drugs on Metabolite Profiles in Rat Brain Using ^1^ H NMR Spectroscopy. J. Proteome Res..

[103] Tkachev D., Mimmack M.L., Huffaker S.J., Ryan M., Bahn S. (2007). Further evidence for altered myelin biosynthesis and glutamatergic dysfunction in schizophrenia. Int. J. Neuropsychopharmacol..

[104] Hodson M.P., Dear G.J., Roberts A.D., Haylock C.L., Ball R.J., Plumb R.S., Stumpf C.L., Griffin J.L., Haselden J.N. (2007). A gender-specific discriminator in Sprague–Dawley rat urine: The deployment of a metabolic profiling strategy for biomarker discovery and identification. Anal. Biochem..

[105] Slupsky C.M., Rankin K.N., Wagner J., Fu H., Chang D., Weljie A.M., Saude E.J., Lix B., Adamko D.J., Shah S. (2007). Investigations of the Effects of Gender, Diurnal Variation, and Age in Human Urinary Metabolomic Profiles. Anal. Chem..

[106] Cynober L.A. (2002). Plasma amino acid levels with a note on membrane transport: Characteristics, regulation, and metabolic significance. Nutrition.

[107] Cho I., Blaser M.J. (2012). The human microbiome: At the interface of health and disease. Nat. Rev. Genet..

[108] Faith J.J., Rey F.E., O’Donnell D., Karlsson M., McNulty N.P., Kallstrom G., Goodman A.L., Gordon J.I. (2010). Creating and characterizing communities of human gut microbes in gnotobiotic mice. ISME J..

[109] Mortha A., Chudnovskiy A., Hashimoto D., Bogunovic M., Spencer S.P., Belkaid Y., Merad M. (2014). Microbiota-Dependent Crosstalk Between Macrophages and ILC3 Promotes Intestinal Homeostasis. Science.

[110] Zhang D., Chen G., Manwani D., Mortha A., Xu C., Faith J.J., Burk R.D., Kunisaki Y., Jang J.-E., Scheiermann C. (2015). Neutrophil ageing is regulated by the microbiome. Nature.

[111] Salgado J.V., Sandner G. (2013). A critical overview of animal models of psychiatric disorders: Challenges and perspectives. Rev. Bras. Psiquiatr..

[112] Donaldson Z.R., Hen R. (2015). From Psychiatric Disorders to Animal Models: A Bidirectional and Dimensional Approach. Biol. Psychiatry.

[113] Zaragoza C., Gomez-Guerrero C., Martin-Ventura J.L., Blanco-Colio L., Lavin B., Mallavia B., Tarin C., Mas S., Ortiz A., Egido J. (2011). Animal Models of Cardiovascular Diseases. J. Biomed. Biotechnol..

